# Determining the nature of the gap in semiconducting graphene

**DOI:** 10.1038/srep41713

**Published:** 2017-02-09

**Authors:** J. C. Prestigiacomo, A. Nath, M. S. Osofsky, S. C. Hernández, V. D. Wheeler, S. G. Walton, D. K. Gaskill

**Affiliations:** 1 Naval Research Laboratory, Washington, DC, USA; 2 George Mason University, Fairfax, VA, USA.

## Abstract

Since its discovery, graphene has held great promise as a two-dimensional (2D) metal with massless carriers and, thus, extremely high-mobility that is due to the character of the band structure that results in the so-called Dirac cone for the ideal, perfectly ordered crystal structure. This promise has led to only limited electronic device applications due to the lack of an energy gap which prevents the formation of conventional device geometries. Thus, several schemes for inducing a semiconductor band gap in graphene have been explored. These methods do result in samples whose resistivity increases with decreasing temperature, similar to the temperature dependence of a semiconductor. However, this temperature dependence can also be caused by highly diffusive transport that, in highly disordered materials, is caused by Anderson-Mott localization and which is not desirable for conventional device applications. In this letter, we demonstrate that in the diffusive case, the conventional description of the insulating state is inadequate and demonstrate a method for determining whether such transport behavior is due to a conventional semiconductor band gap.

The drive for the development of electronic devices using graphene has led to theoretical and experimental studies of methods for inducing a semiconductor gap in graphene. These schemes include exposure to reactive gas backgrounds and/or plasmas to alter surface chemistry^1^,^2^,^3^, the inclusion of dopants in the lattice^4^, the quantum confinement of electrons in nanoribbons^5^, and the dual-gating of graphene bilayers^6^. The presence of a gap is experimentally determined through electrical transport measurements, where the resistivity is expected to exhibit thermally activated behavior that increases with decreasing temperature according to an Arrhenius law, or directly through photo-emission spectroscopy.

Previous work has shown that the transport properties of graphene are sensitive to disorder. It is known that weak-localization (WL) and enhanced electron-electron interactions (EEI) control the metallic transport properties in disordered conductors. For metallic graphene with moderately high mobility, there have been several studies reporting WL and/or EEI^7^,^8^,^9^,^10^,^11^,^12^,^13^,^14^,^15^. Often, these phenomena manifest as a change from conventional metallic (dR/dT > 0) to activated (dR/dT < 0) behaviors at low temperatures. Furthermore, Chen, *et al*. have demonstrated that insulating samples result through exposure to ion damage^16^. Bostwick, *et al*. observed a large increase in room temperature resistance accompanied by a breakdown of the quasi-particle description as determined from photoemission in graphene exposed to atomic hydrogen^17^. Moreover, reports of Arrhenius transport at the Dirac point are always accompanied by strong-localization effects, with a dominant resistivity component that follows Mott’s variable range hopping law^6^,^18^. Thus, it is clear that disordered graphene will exhibit activated resistivity behavior that mimics that of graphene with a band gap.

In this report, we present transport data for graphene samples that were chemically modified via surface functionalization by exposure to low energy plasmas to induce a metal-insulator transition. These results show that increasing resistivity with decreasing temperature is not an adequate metric for determining whether a conventional band gap, and thus, band semiconductor behavior, is present in graphene. We also show that the temperature dependence of the mobility along with the presence of variable range hopping can be used to determine whether activated resistivity is caused by the presence of disorder rather than the existence of a band gap.

The key to distinguishing between the two types of activated behavior is to contrast the scattering processes that influence transport in the two regimes. For high mobility conductors and semiconductors (which are narrow gap band insulators), the conductivity can be described by the familiar Drude expression:


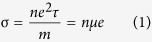


where *n* is the carrier concentration, *e* is the electron charge, *τ* is the relaxation time for the dominant scattering mechanism, *m* is the effective electron mass, and *μ* is the mobility. Use of this expression is predicated on the assumption that the material is well ordered with a mean free path that is much longer than the inverse of the magnitude of the Fermi wave number (k_F_l ≫ 1). For applicable conductors, the high temperature conductivity is controlled by phonon scattering with the relaxation time, and hence the conductivity, increasing (i.e. decreasing resistivity) with decreasing temperature. At low temperature, temperature dependent scattering mechanisms become negligible (For this description, we neglect electron-electron scattering which alters the temperature dependence of σ at low temperature) and static impurity scattering dominates, leaving the conductivity temperature independent with a *μ* that increases with decreasing temperature before saturating to a constant value. For a semiconductor, the Fermi energy falls within the band gap, resulting in an *n* and σ that exponentially decrease with decreasing temperature. Thus, for this situation, R ~ exp(1/T) and *μ* again increases with decreasing temperature before saturating at a constant value^19^. As the disorder of the material is increased, the mean free path decreases so that equation (1) is no longer the proper description for transport. Since transport in this case is diffusive, conductivity is described by:


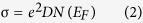


with the diffusivity, 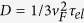 where *τ*_*el*_ is the elastic scattering time, and with the density of states at the Fermi energy, *N(E*_*F*_). In this formulation the mobility is 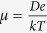. For metals, the effects of this strong disorder manifest as an increase in the low temperature resistivity due to weak-localization and enhanced electron-electron interactions^20^,^21^ and the conventional practice of determining the mobility as the ratio between conductivity and carrier concentration is not valid. Highly disordered materials are strongly localized and are thus insulators with σ = 0 at T = 0. Transport in these insulators are described by variable range hopping (VRH) with R ~ exp(T^−1/4^) in 3D and R ~ exp(T^−1/3^) in 2D. Therefore, it is necessary to examine the temperature dependences of the resistivity and mobility to determine whether a material is a band or disordered insulator.

Epitaxial graphene samples for this study were grown via Si sublimation from nominally on-axis SiC (0001) substrates as described elsewhere^22^. These conditions resulted in graphene with an average thickness of 1.5 layers as determined by x-ray photoelectron spectroscopy. The samples were then fashioned into a pattern that enabled standard four-probe resistivity and Hall measurements^23^. Each sample was then systematically exposed for 6 s to electron beam generated plasmas produced in 50 to 90 mTorr mixtures of O_2_ or N_2_ to introduce oxygen- or nitrogen-functional groups^24^,^25^,^26^. A total of three N_2_ doses were administered to the nitrogen-functionalized samples while two O_2_ doses plus a final vacuum anneal were performed on the O_2_ functionalized sample.

Raman spectroscopy and XPS were performed both before and after functionalization (see Supplementary information of ref. 23 for details) in order to ensure that the sp^2^ nature of the graphene was recoverable after functionalization and vacuum annealing and that the integrity of its interfacial buffer layer remained intact. The Raman spectra confirmed that the characteristic 2D peak of graphene was present after each plasma dose and, as expected, a disorder-induced D peak emerged after the first dose that increased in intensity with increasing dosage. Furthermore, a peak in the XPS spectra associated with the interface layer was observed before functionalization and again after vacuum annealing.

As prepared, the resistance of the graphene samples at high temperatures behaves like a conventional metal (Fig. 1a,b). Below ~50 K the resistance exhibits an upturn and increases with decreasing temperature. This behavior has been observed previously^6^,^7^,^8^,^9^,^10^,^11^,^12^,^13^,^14^, and is due to WL and/or EEI caused by disorder. The temperature dependent mobilities were determined from the conductivity and Hall resistance, R_Hall_, measurements in the conventional manner (Fig. 1a,b). As is easily seen in Fig. 1a and b, the mobility appears to decrease below 50 K concurrently with the increase in resistance, and because the resistance increase is known to be due to disorder effects, we conclude that the anomalous low temperature behavior of the calculated mobility is due to the inapplicability of equation (1) in this temperature range. Thus, we conclude that when WL and/or EEI effects are apparent in the resistance, mobility as determined in the conventional manner is meaningless and that semi-classical mechanisms for explaining deceasing mobility with decreasing temperature, such as ionized impurity scattering^19^,^27^, are inappropriate.

After the initial electrical characterization, the samples were subjected to a series of low energy plasma exposures and again characterized^22^. The higher exposures transformed the samples into insulators (examples are shown in Fig. 2a,b). For these samples the temperature dependence of the conventionally calculated mobilities are again anomalous with behaviors ranging from continuously decreasing with decreasing temperature (Fig. 1b) to increasing with decreasing temperature with a turnover at intermediate temperatures (Fig. 1a). We again infer that the anomalous temperature dependence of the mobilities is due to the inapplicability of equation (1), this time for the semiconducting (or insulating) state. To further explore the nature of transport in these samples we plotted log(R) versus 1/T, the form expected for conventional semiconductors (Fig. 3a,b). These curves show that the samples do not obey the expected behavior of conventional semiconductors, even at low temperatures. In Fig. 4a,b, the same data are plotted versus T^−1/3^, the temperature dependence expected for 2D VRH. These results clearly show that the temperature dependence of the resistance is consistent with 2D VRH, especially at low temperature, thus showing that chemical functionalization, using this approach, does not produce a band gap in graphene.

In conclusion, we have shown that plasma functionalized graphene exhibits thermally activated resistance that, at first glance appears to show semiconducting behavior. Careful examination of the temperature dependence of the resistance and mobility reveals that these quantities do not have the temperature dependences expected for band semiconductors with the mobility decreasing with decreasing temperature for at least some temperature intervals below 300 K and instead the resistance shows a 2D variable range hopping behavior. Importantly, these results illustrate a method for determining whether graphene processed to produce a band gap for electronic device applications are truly conventional band semiconductors.

## Additional Information

**How to cite this article:** Prestigiacomo, J. C. *et al*. Determining the nature of the gap in semiconducting graphene. *Sci. Rep.* **7**, 41713; doi: 10.1038/srep41713 (2017).

**Publisher's note:** Springer Nature remains neutral with regard to jurisdictional claims in published maps and institutional affiliations.

## Figures and Tables

**Figure 1:**
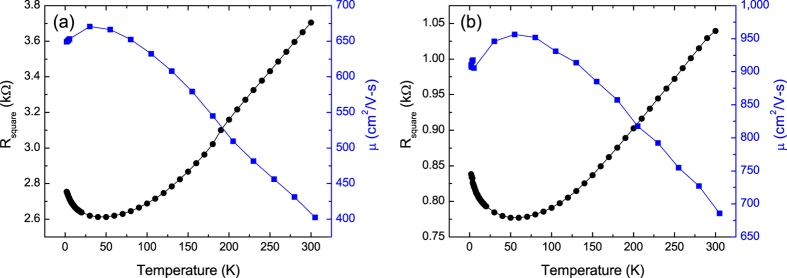
Temperature dependence of the resistance and mobility for unfunctionalized graphene samples.

**Figure 2:**
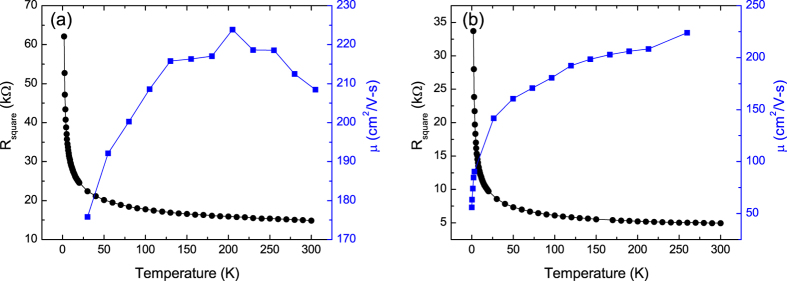
Temperature dependence of the resistance and mobility for samples functionalized with (**a**) nitrogen and (**b**) oxygen plasmas.

**Figure 3:**
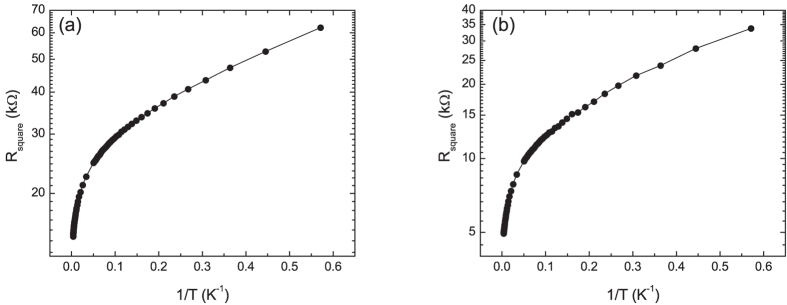
Plot of log(R) vs 1/T for samples functionalized with (**a**) nitrogen and (**b**) oxygen plasmas.

**Figure 4:**
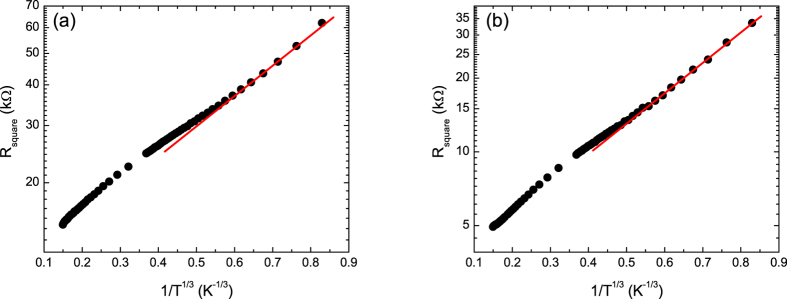
Plot of log(R) vs 1/T^1/3^ for samples functionalized with (**a**) nitrogen and (**b**) oxygen plasmas. The solid lines are guides to the eye.
